# Immunity against *Neisseria meningitidis* Serogroup C in the Dutch Population before and after Introduction of the Meningococcal C Conjugate Vaccine

**DOI:** 10.1371/journal.pone.0012144

**Published:** 2010-08-13

**Authors:** Richarda M. de Voer, Liesbeth Mollema, Rutger M. Schepp, Sabine C. de Greeff, Pieter G. M. van Gageldonk, Hester E. de Melker, Elisabeth A. M. Sanders, Guy A. M. Berbers, Fiona R. M. van der Klis

**Affiliations:** 1 Laboratory for Infectious Diseases and Screening, National Institute of Public Health and the Environment, Bilthoven, The Netherlands; 2 Department of Immunology, University Medical Centre, Utrecht, The Netherlands; 3 Epidemiology and Surveillance Unit, National Institute for Public Health and the Environment, Bilthoven, The Netherlands; 4 Department of Paediatric Immunology and Infectious Diseases, University Medical Centre Utrecht, Utrecht, The Netherlands; Nuffield Orthopaedic Centre, United Kingdom

## Abstract

**Background:**

In 2002 a Meningococcal serogroup C (MenC) conjugate vaccine, with tetanus toxoid as carrier protein, was introduced in the Netherlands as a single-dose at 14 months of age. A catch-up campaign was performed targeting all individuals aged 14 months to 18 years. We determined the MenC-specific immunity before and after introduction of the MenC conjugate (MenCC) vaccine.

**Methods and Findings:**

Two cross-sectional population-based serum banks, collected in 1995/1996 (*n* = 8539) and in 2006/2007 (*n* = 6386), were used for this study. The main outcome measurements were the levels of MenC polysaccharide(PS)-specific IgG and serum bactericidal antibodies (SBA) after routine immunization, 4–5 years after catch-up immunization or by natural immunity. There was an increasing persistence of PS-specific IgG and SBA with age in the catch-up immunized cohorts 4–5 years after their MenCC immunization (MenC PS-specific IgG, 0.25 µg/ml (95%CI: 0.19–0.31 µg/ml) at age 6 years, gradually increasing to 2.34 µg/ml,(95%CI: 1.70–3.32 µg/ml) at age 21–22 years). A comparable pattern was found for antibodies against the carrier protein in children immunized above 9 years of age. In case of vaccination before the age of 5 years, PS-specific IgG was rapidly lost. For all age-cohorts together, SBA seroprevalence (≥8) increased from 19.7% to 43.0% in the pre- and post-MenC introduction eras, respectively. In non-immunized adults the SBA seroprevalence was not significantly different between the pre- and post-MenC introduction periods, whereas PS-specific IgG was significantly lower in the post-MenC vaccination (GMT, age ≥25 years, 0.10 µg/ml) era compared to the pre-vaccination (GMT, age ≥25 years, 0.43 µg/ml) era.

**Conclusion:**

MenCC vaccination administered above 5 years of age induced high IgG levels compared to natural exposure, increasing with age. In children below 14 months of age and non-immunized cohorts lower IgG levels were observed compared to the pre-vaccination era, whereas functional levels remained similar in adults. Whether the lower IgG poses individuals at increased risk for MenC disease should be carefully monitored. Large-scale introduction of a MenCC vaccine has led to improved protection in adolescents, but in infants a single-dose schedule may not provide sufficient protection on the long-term and therefore a booster-dose early in adolescence should be considered.

## Introduction

In September 2002, a single Meningococcal serogroup C conjugate (MenCC, Neisvac-C, Baxter, IL, USA) vaccination at the age of 14 months was introduced for all newborns in the Dutch national immunization programme (NIP). The reason to include MenCC vaccination in the NIP was the rapidly progressive increase in the incidence of MenC disease in 2000–2001 [1]. The decision for a single dose schedule at 14 months of age was based on epidemiological, programmatical and economical reasons [2]. Next to vaccination of all 14-month-old children, a catch-up campaign was conducted between June and November 2002 for all children and adolescents between 1 and 18 years of age, who were invited to receive a single MenCC dose (overall vaccine coverage 94%) [3]. Soon afterwards, MenC disease disappeared in vaccinated persons, and a sharp decline was observed in non-immunized cohorts [1].

Whereas the Netherlands had introduced a single injection at 14 month of age and a catch-up campaign, different MenCC vaccine schedules were introduced within Europe. The United Kingdom and Spain implemented a 3-dose primary series at 2, 3 and 4 months and 2, 4 and 6 months of age, respectively. However, already within the first year after the last scheduled dose in infancy low effectiveness was observed [4], [5]. This was explained by rapidly waning antibody titers after vaccinations early in life without a later booster. Afterwards, the UK changed the vaccination schedule to 2 priming doses at 3 and 4 months of age followed by a booster dose at 12 months [6]. Rapid waning of circulating antibodies was also observed in the UK after a single dose in the second year of life [7], [8]. In contrast, when MenCC vaccine was administered at older ages, between 6 and 18 years, a single vaccination, resulted in persistently high antibody levels and (bactericidal) antibody levels up to at least five years after vaccination [9], [10].

In the Netherlands, no vaccine failures have been reported and only sporadic cases of MenC disease in non-immunized age-cohorts have occurred, indicating low transmission due to ongoing herd-effects after introduction of vaccination. However, monitoring the persistence of vaccine-induced protection in various age categories after a single immunization remains relevant since widespread introduction of the conjugate vaccine has led to reduced circulation, leading to a lack in natural boosting, eventually resulting in possible waning immunity in both vaccinated and non-vaccinated age-cohorts.

A widely accepted correlate of protection for MenC disease is the outcome of a serum bactericidal antibody (SBA) assay [11], [12]. MenC polysaccharide-specific immunoglobulin type G (IgG) responses may provide insight in naturally-acquired or vaccine-induced immunity [10], [13], [14]. Here we describe the seroprevalence of MenC polysaccharide-specific antibody concentrations and functional SBA titres in two cross-sectional population-based serum sets, collected several years before (1995/6) and after introduction (2006/7) of the MenCC vaccine in 2002. Furthermore, the MenCC vaccine administered in the Dutch NIP contains tetanus toxoïd as the carrier protein. For this reason we also examined antibody levels directed towards tetanus in age-cohorts who received a MenCC immunization during the catch-up campaign in 2002.

## Methods

The study proposal was approved by the Medical Ethics Testing Committee of the foundation of therapeutic evaluation of medicines (METC-STEG) in Almere (clinical trial number: ISRCTN 20164309) and all participants provided signed informed consent.

### Study population

Two independent cross-sectional population-based serosurveillance studies were carried out in the Netherlands between October 1995- December 1996 (*n* = 8539) and February 2006- June 2007 (*n* = 6386) (ISRCTN 20164309). Both studies had the same design which has been described previously [15], [16]. Briefly, a sample of eight municipalities was drawn proportional to the number of inhabitants in five geographical regions of approximately equal population size in the Netherlands. Within each municipality an age-stratified sample (0, 1–4, 5–9, . . ., 75–79 years) of 380–500 persons (males and females) was drawn. The first two age strata were over-sampled due to an expected lower response rate in these groups. In total 17,341 persons were invited in the nationwide sample. An extra sample of non-Western migrants was taken from 12 of the 40 municipalities in the nationwide sample. In total 2,574 migrants were invited. Eligible individuals were requested to donate a blood sample, to complete a questionnaire (questions regarding demographic characteristics, vaccination history, health perception and diseases, and activities possibly related to infectious diseases (e.g. travelling, profession, gardening)), and to provide immunization records. The immunization history of the participants was checked by copies of immunization certificates and information obtained from regional immunization administration office archives. All participants gave signed informed consent. Collected serum samples were stored at −80°C until analysis.

From the pre-immunization era, 2303 sera were randomly selected and tested for detection of MenC-specific IgG, and from these samples 735 were randomly selected (equally distributed over age and blinded to the IgG result) and tested for MenC-specific SBA. Sufficient amount of serum was available for 6376 samples from the post-immunization era and these were tested for meningococcal serogroup A−, C−, Y− and W-135-specific IgG and from these samples 1220 were randomly selected (again equally distributed over age and blinded to IgG result) and tested for MenC-specific SBA. Laboratory staff was blinded to sample characteristics (e.g. age, gender, et cetera) during analysis and all analyses were performed in 2007/8.

### Laboratory methods

Meningococcal polysaccharide-specific IgG was determined by a fluorescent-bead-based multiplex immunoassay (MIA) as previously described [17]. Tetanus-specific antibodies were determined with a MIA as described previously by Van Gageldonk et al [18]. Samples were analyzed using a Bio-Plex system in combination with the Bio-Plex Manager software version 4.1.1 (Bio-Rad Laboratories, Hercules, CA). For each analyte, median fluorescent intensity was converted to µg/ml (meningococcal serogroups) or international units (IU)/ml (tetanus) by interpolation from a 5-parameter logistic standard curve. A concentration of 2 µg/ml was used as cut-off in the MIA, which is internationally accepted as standard [13], [14]. The lower limit of quantitation for all four meningococcal serogroups was assigned at 0.01 µg/ml and for tetanus at 0.005 IU/ml for statistical purposes.

The level of MenC-specific functional antibodies was determined by a serum bactericidal antibody assay (SBA) using baby rabbit complement [19], and the *O*-acetylated serogroup C strain C11 (phenotype C:16:P1.7–1,1). SBA titres were expressed as the reciprocal of the final serum dilution yielding ≥50% killing at 60 min. A titer of 8 was used as cut-off in the SBA, which is internationally accepted as standard [10], [11], [12]. SBA titres <4 were assigned a value of 2 for statistical purposes. The SBA measurements were performed as a post-hoc analysis to the original protocol.

### Statistical analysis

Data analyses were performed in SAS 9.1.3 (SAS Institute Inc. Cary, NC, USA). Seroprevalences and geometric mean concentrations (GMC) or geometric mean titers (GMT) of meningococcal serogroup-specific IgG and serum bactericidal activity, respectively, were calculated for the pre- and postvaccination survey in different age-cohorts and weighted. In addition, GMCs of tetanus specific antibodies were calculated and weighted. Weights were determined proportional to the reference population (Dutch population, 1^st^ of January 1997 for the pre-MenC introduction study and 1^st^ of January 2007 for the post-MenC introduction study), taking into account sex and age. For the post-MenC vaccination study the degree of urbanization and ethnicity were also taken into account because of the additional sample of non-Western migrants, which was analyzed together with the nationwide sample. The stratified age-bands from the original protocol were adjusted post-hoc, to enable a better separation of the age-cohorts with respect to their MenC immunization status. Age-specific differences in MenC IgG levels between the pre- and postvaccination surveys in each age-cohort were determined by a Wilcoxon two-sided Z-test. Likewise, differences in MenC-specific SBA seroprevalence (antibody titers ≥8) were determined by a chi-square distribution (LR chi-square probability) or two-sided Fisher's exact test, as appropriate. No multiplicity was taken into account since only pre- and post-MenC introduction data within each age-cohort were compared and the age-cohorts were not mutually compared. The Spearman rank correlation was used to estimate the correlation between age-specific IgG levels and SBA seroprevalence. *P*-values of <0.05 were considered statistically significant.

## Results

### Study characteristics

Samples from the pre-MenC introduction study were collected between October 1995 and December 1996, median 6.6 (SD, 0.3) years before introduction of MenCC vaccination in the Netherlands in 2002. Samples from the post-MenC introduction era were collected between February 2006 and June 2007, a median of 4.3 years (SD, 0.4) after widespread implementation of MenCC vaccination in 2002. MenC immunization dates were available for 82% (*n* = 1273) of the individuals in cohorts eligible for MenCC vaccination: either by routine immunization at 14 months of age or during the catch-up campaign in 2002.

### MenC-specific antibody levels in the pre-MenC introduction era

**Figure 1:**
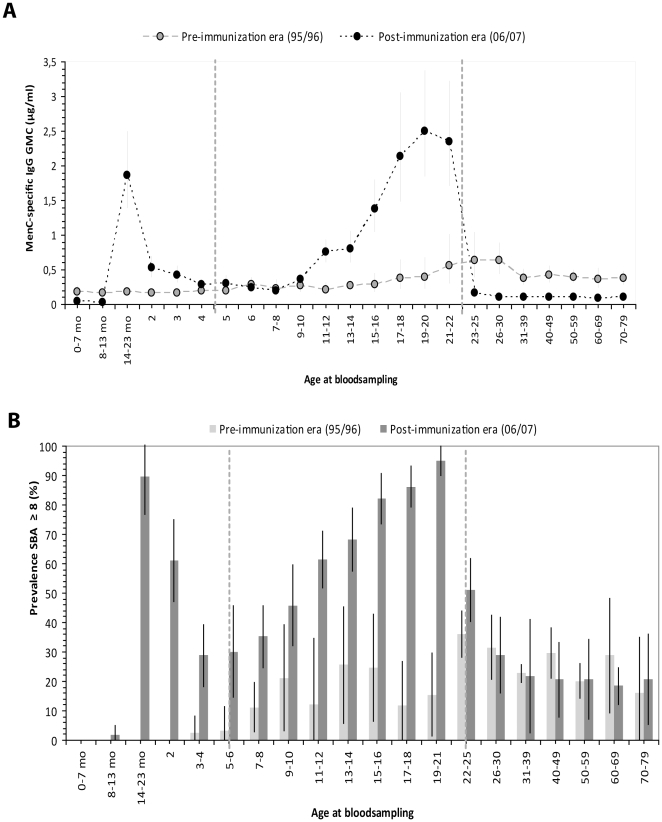
MenC-specific IgG and serum bactericidal antibody levels. MenC PS-specific IgG (A) and seroprevalence of SBA titers ≥8 (B) within each age-cohort, pre- and post-introduction of the MenC conjugate vaccine. Error bars indicate 95% confidence intervals. Between the vertical lines cohorts are indicated that were all immunized in the catch-up campaign of 2002. Age at bloodsampling is indicated in years or as stated otherwise (mo  =  age in months).

Before introduction of the MenCC vaccine, in general, low MenC-specific IgG levels were observed, with an overall GMC of 0.37 µg/ml. The lowest levels were observed under 5 years of age (GMC 0.18 µg/ml, ages 0–5 years). After the age of 11–12 years, a small increase in IgG was observed (GMC 0.22 µg/ml, at 11–12 years of age) until the age-cohort of 26–30 years (GMC 0.63 µg/ml), after which antibody levels stabilized at a lower level **(** **Fig. 1A** **,** **Table 1** **)**. The prevalence of SBA titers ≥8 in the pre-MenC introduction era followed a similar pattern. A low prevalence of functional SBA titers under 6 years of age, hereafter, the prevalence of putative protective SBA levels increased from 11% at the ages 9–10 years of age to 26% at the ages 22–25 years. In cohorts over 22 years of age on average 23% revealed SBA titers ≥8 **(** **Fig. 1B** **,** **Table 2** **)**.

**Table 1:** Number of samples within each age-cohort, GMCs and seroprevalence of MenC PS-specific IgG (≥2 µg/ml) in the pre- and post-MenC introduction eras.

Age	Pre-introduction MenCC vaccine			Post-introduction MenCC vaccine			*P*-value*
	No. samples	GMC (95% CI)	% samples ≥2 µg/ml	No. samples	GMC (95% CI)	% samples ≥2 µg/ml	Pre vs Post GMC
0–7 mo	35	0.17 (0.12−0.24)	2.5 (0.00−7.63)	202	0.04 (0.03−0.05)	0.2 (0.00−0.50)	<.0001
8–13 mo	98	0.16 (0.13−0.20)	0.9 (0.00−2.67)	163	0.03 (0.02−0.04)	0 (NA)	<.0001
14–23 mo	77	0.18 (0.15−0.20)	0.0 (NA)	88	1.87 (1.39−2.51)	58.8 (48.13−69.38)	<.0001
2 y	108	0.16 (0.14−0.20)	0.9 (0.00−2.63)	116	0.53 (0.39−0.71)	12.5 (6.09−18.93)	<.0001
3 y	123	0.16 (0.14−0.19)	2.4 (0.00−5.02)	142	0.43 (0.33−0.57)	13.8 (8.39−19.14)	<.0001
4 y	100	0.19 (0.16−0.23)	1.1 (0.00−3.29)	145	0.29 (0.23−0.37)	5.5 (1.23−9.73)	<.0001
5 y	80	0.20 (0.15−0.26)	4.9 (0.00−9.80)	95	0.31 (0.25−0.38)	9.1 (4.71−13.59	0.0106
6 y	84	0.27 (0.19−0.38)	13.0 (4.39−21.67)	107	0.25 (0.19−0.31)	4.4 (0.05−8.75)	0.2374
7–8 y	58	0.22 (0.14−0.35)	6.9 (0.00−16.15)	257	0.20 (0.16−0.26)	5.4 (3.13−7.67)	0.0527
9–10 y	49	0.28 (0.18−0.44)	12.4 (2.18−22.62)	244	0.36 (0.26−0.51)	8.5 (4.73−12.26)	0.0083
11–12 y	62	0.22 (0.15−0.31)	9.6 (1.30−17.85)	188	0.76 (0.61−0.94)	27.0 (20.75−33.36)	<.0001
13–14 y	73	0.28 (0.21−0.36)	14.1 (6.33−21.94)	159	0.80 (0.61−1.06)	28.6 (20.83−36.44)	<.0001
15–16 y	74	0.30 (0.19−0.47)	18.7 (8.98−28.44)	138	1.37 (1.06−1.77)	39.0 (30.98−46.99)	<.0001
17–18 y	61	0.40 (0.23−0.67)	19.3 (7.26−31.37)	113	2.14 (1.49−3.07)	45.9 (36.50−55.36)	<.0001
19–20 y	44	0.40 (0.23−0.69)	18.7 (7.07−30.31)	148	2.50(1.86−3.35)	49.6 (41.98−57.20)	<.0001
21–22 y	30	0.56 (0.31−1.01)	31.2 (13.8−48.53)	137	2.34 (1.70−3.23)	50.5 (43.08−57.82)	0.0003
23–25 y	70	0.64 (0.39−1.06)	26.1 (15.5−36.74)	185	0.16 (0.11−0.23)	16.0 (9.78−22.15)	0.0001
26–30 y	116	0.63 (0.45−0.88)	29.1 (20.45−37.70)	346	0.10 (0.08−0.13)	9.7 (6.02−13.34)	<.0001
31–39 y	205	0.38 (0.28−0.52)	15.3 (9.64−21.00)	658	0.11 (0.09−0.13)	9.9 (7.29−12.59)	<.0001
40–49 y	203	0.43 (0.32−0.56)	20.9 (14.78−27.05)	641	0.11 (0.09−0.13)	8.3 (6.27−10.41)	<.0001
50–59 y	202	0.40 (0.32−0.50)	15.7 (9.84−21.54)	714	0.10 (0.09−0.11)	5.9 (3.92–7.96)	<.0001
60–69 y	182	0.37 (0.25−0.55)	14.7(9.04−20.39)	798	0.08 (0.07−0.10)	4.2 (2.26−6.16)	<.0001
70–79 y	171	0.38 (0.29−0.49)	13.0 (7.44−18.66)	592	0.10 (0.09−0.11)	6.8 (4.81−8.81)	<.0001
Total	2305	0.37 (0.32−0.44)	16.8 (14.2−19.4)	6376	0.17 (0.16−0.18)	12.6 (11.7−13.6)	<.0001

*P-values calculated for differences between IgG GMC pre- and post-introduction of the MenC vaccine with the Wilcoxon two-sided Z-test.

GMC, geometric mean concentration; NA, not applicable; mo, age in months; y, age in years.

**Table 2:** Number of samples within each age-cohort, GMTs and seroprevalence of SBA (titers ≥8) in the pre- and post-MenC introduction eras.

Age	Pre-introduction MenCC vaccine			Post-introduction MenCC vaccine			*P*-value*
	No. samples^a^	GMT (95% CI)	% samples ≥8	No. samples	GMT (95% CI)	% samples ≥8	Pre vs Post % ≥8
0–7 mo	3	2 (NA)	0 (NA)	59	2 (NA)	0 (NA)	NP
8–13 mo	13	2.1 (1.9−2.4)	0 (NA)	60	2.1 (1.9−2.4)	1.7 (0.00−4.90)	0.6814**
14–23 mo	18	2.0 (NA)	0 (NA)	29	122.0 (59.9−248.6)	89.7 (79.04−100.00)	<.0001
2 y	24	2.0 (NA)	0 (NA)	42	13.1 (8.3−20.8)	61.9 (49.24−74.57)	<.0001
3–4 y	42	2.1 (1.9−2.4)	2.6 (0.00−8.78)	106	5.4 (3.8−7.7)	30.2 (19.76−40.62)	<.0001
5–6 y	34	2.8 (1.6−4.7)	6 (0.00−16.38)	49	5.2 (3.4−7.8)	28.6 (14.44−42.70)	0.0061
7–8 y	43	3.4 (2.2−5.2)	11.2 (2.73−19.58)	56	5.1 (3.7−7.1)	33.9(23.18−44.68)	0.0081
9–10 y	27 (30) a	3.0 (1.3−6.9)	11.3 (0.00−28.38)	73	9.4 (5.8−15.2)	45.2 (31.66−58.75)	0.0135
11–12 y	31 (32) a	2.5 (1.4−4.5)	8.4 (0.00−29.35)	72	20.0 (12.4−32.2)	61.1 (51.19−71.03)	<.0001
13–14 y	34 (35) a	5.6 (2.1−14.8)	23.5 (3.23−43.83)	65	23.5 (15.9−34.6)	69.2 (59.20−79.26)	<.0001
15–16 y	38 (42) a	3.3 (2.1−5.1)	16.3 (0.00−35.54)	55	57.9 (36.7−91.1)	81.8 (72.92−90.71)	<.0001
17–18 y	25	3.6 (1.8−7.1)	11.7 (0.00−26.93)	43	89.8 (58.4−138.0)	86 (78.87−93.22)	<.0002
19–21 y	21 (23) a	2.6 (1.9−3.5)	5.9 (0.00−14.56)	88	159.6 (109.1−233.4)	95.5 (90.86−100.00)	<.0001**
22–25 y	38 (44) a	6.2 (3.1−12.4)	26.1 (10.90−41.30)	104	14.4 (9.1−22.6)	49.0 (38.70−59.38)	0.092
26–30 y	56 (62) a	4.6 (2.1−10.0)	24.5(8.76−40.30)	69	4.2 (3.1−5.9)	26.1 (13.14−39.03)	0.563
31–39 y	66 (67) a	4.7 (3.8−5.8)	22.5 (17.98−27.05)	58	3.6 (2.1−6.0)	19.0 (1.32−36.61)	0.6376
40–49 y	65 (69) a	5.3 (4.0−6.9)	26 (20.78−31.30)	49	4.1 (2.5−6.7)	20.4 (8.28−32.54)	0.2877
50–59 y	59	4.5 (3.9−5.2)	19.2 (13.64−24.75)	52	4.1 (2.5−6.6)	21.2 (6.14−36.17)	0.9105
60–69 y	51	5.9 (3.0−11.5)	29.5 (11.85−47.24)	54	3.2 (2.7−3.7)	18.5 (12.33−24.71)	0.1894
70–79 y	48	3.6 (2.1−6.1)	17.1 (0.00−37.18)	37	3.6 (2.5−5.4)	21.6 (6.11−37.14)	0.7433
Total	736 (764) a	4.3 (3.3−5.5)	19.8 (13.7−26.0)	1220	10.2 (8.9−11.7)	43.0 (39.5−46.5)	<.0001

a Number between brackets is the number of samples included in the seroprevalence calculation (% samples≥8) and in not the GMT calculation, because only a SBA titer ≥16 could be given for these samples.

*P-values calculated for differences between prevalence of SBA titers ≥8 pre- and post-MenC immunization with chi-square distribution, LR chi-square probability.

**Calculated with Fishers exact t-test.

GMT, geometric mean titer; NP, not possible; NA, not applicable; mo, age in months; y, age in years.

### MenC specific antibody levels in the post-MenC introduction era

After implementation of routine MenCC vaccination at 14 months of age in 2002, MenC-specific IgG levels under 14 months of age were significantly lower in 2006/2007 compared to the levels in 1996/1997 (*P*<0.0001*)* **(** **Fig. 1A** **,** **Table 1** **)**. None of the tested sera of infants under 7 months and only 3% of those between 8 and 14 months of age revealed putative protective SBA levels ≥8 in the post-MenC introduction era. From the pre-MenC introduction era only a few samples of infants under 14 months (*n* = 16) were available for SBA testing and none of these samples revealed a SBA titer ≥8.

After introduction of vaccination, a clear rise in MenC-specific IgG and functional antibodies was observed in the age-cohort 15–23 months **(** **Fig. 1A and B** **)**. In accordance, the prevalence of SBA titers ≥8 in infants between 15 and 23 months of age was 92.6% (MenC-specific IgG GMC, 2.43 µg/ml). However, after this peak in antibody levels, a steep decline in IgG antibodies was found **(** **Fig. 1A** **,** **Table 1** **),** and IgG antibody concentrations had decreased to pre-MenC introduction era levels in children between 23 and 72 months of age. SBA levels also had declined in the years following immunization at 14 months but remained well above the SBA levels observed in the pre-introduction era (*P*<0.0001) **(** **Fig. 1B** **,** **Table 2** **)**.

Individuals, who had been vaccinated with a single injection between 14 months and 18 years of age during the catch-up campaign in 2002, were 5 to 22 years of age during the post-MenC introduction study in 2006/7. Children aged 6–9 years in the post-MenC introduction survey revealed the lowest MenC-specific IgG levels (GMC 0.24 µg/ml), which were comparable to the pre-MenC introduction era levels (GMC 0.25 µg/ml) in the same age-cohorts **(** **Fig. 1A** **,** **Table 1** **)**. Nonetheless, the prevalence of functional SBA titers was higher in the age-cohort 6 to 9 years compared with the pre-introduction era (*P*<0.05) **(** **Fig. 1B** **,** **Table 2** **)**. Remarkably, MenC-specific IgG and SBA titers had increased gradually with age for children vaccinated between 5 and 18 years of age and were significantly higher than in the pre-MenC introduction era (*P*<0.0001) **(** **Fig. 1A and B** **)**. IgG levels had increased from 0.36 µg/ml in the cohort 9–10 years of age to a level of 2.34 µg/ml in the ages 21–22 years and the SBA prevalence ≥8 had increased from 45.2% to 95.5% in the age-cohorts 9–10 and 19–21 years, respectively. The prevalence of SBA titers ≥8 in the post-MenC introduction era in cohorts over 25 years of age was again comparable to the pre-MenC introduction era: 21% and 23%, respectively. In non-immunized cohorts aged 25 years and over, MenC-specific IgG levels were significantly lower in the post-MenC introduction era (GMT age ≥25 years, 0.10 µg/ml) compared with pre- introduction (GMT age ≥25 years, 0.43 µg/ml) levels.

Overall, in all age categories together, a higher GMC of MenC-specific IgG was observed before introduction of the MenCC vaccine in 2002 (GMC 0.37 µg/ml), compared with the post-MenC introduction era (GMC 0.17 µg/ml). In contrast, the overall seroprevalence of SBA titers ≥8 was higher after wide-spread introduction of MenC vaccination than before, 43.0% vs 19,7% respectively. The age-specific IgG levels and SBA titers correlated well in the post-MenC introduction era (*R* = 0.72). Before MenC introduction little correlation was observed between IgG and SBA levels (*R* = 0.32).

**Figure 2:**
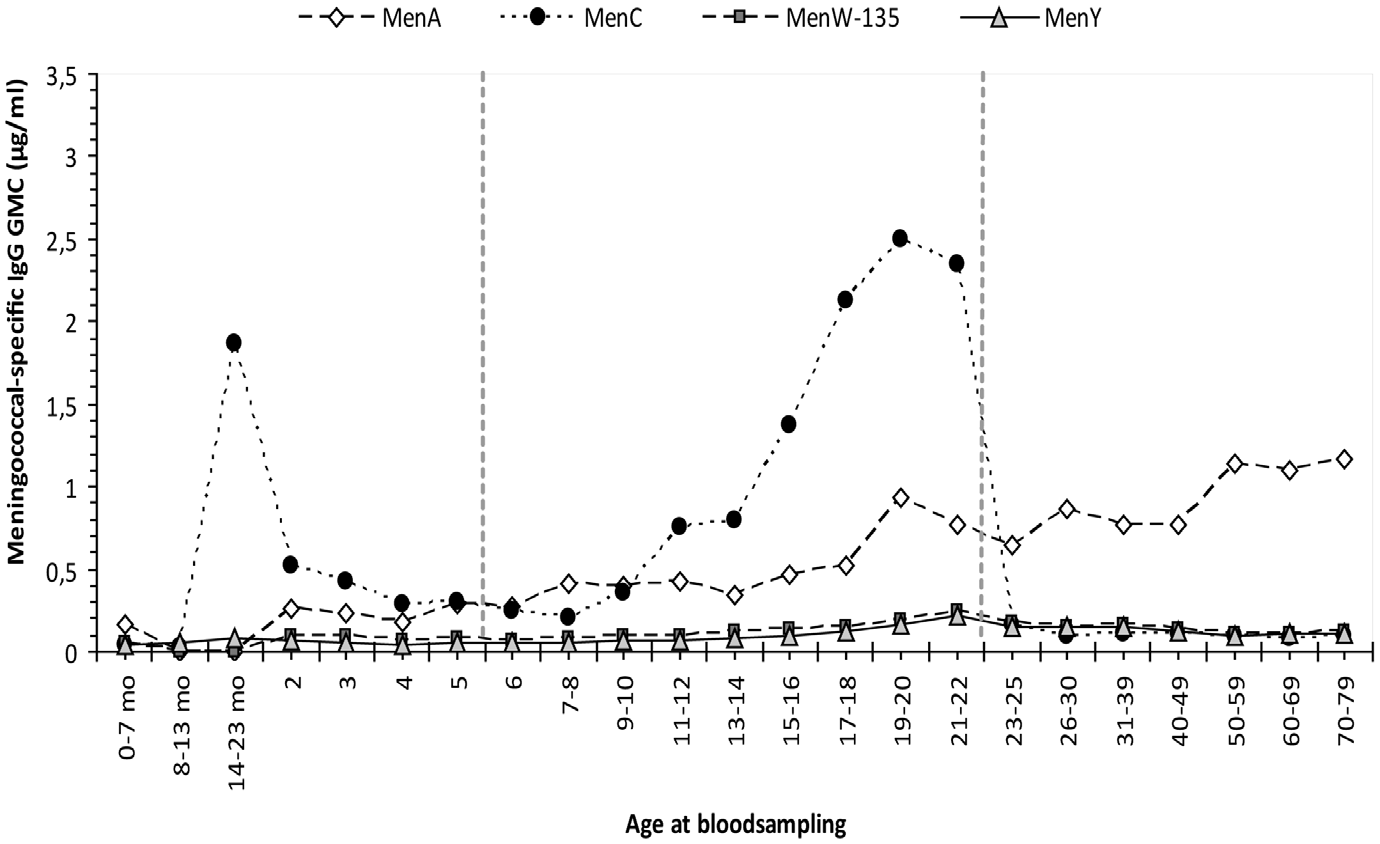
MenACYW-135 -specific IgG antibody levels. MenACYW-135 -specific IgG within each age (group) in the post-MenC introduction era. Age at bloodsampling is indicated in years or as stated otherwise (mo  =  age in months).

With respect to non-MenC IgG antibody levels in the post-MenC introduction era, meningococcal serogroups W-135 and –Y -specific IgG levels were low in all age categories **(** **Fig. 2** **)**. Meningococcal serogroup A-specific IgG however increased with age and was significantly higher than MenC-specific IgG above 25 years of age *(P*<0.0001*)* **(** **Fig. 2** **)**.

### Tetanus-specific antibodies in the pre- and post-MenC introduction eras

**Figure 3:**
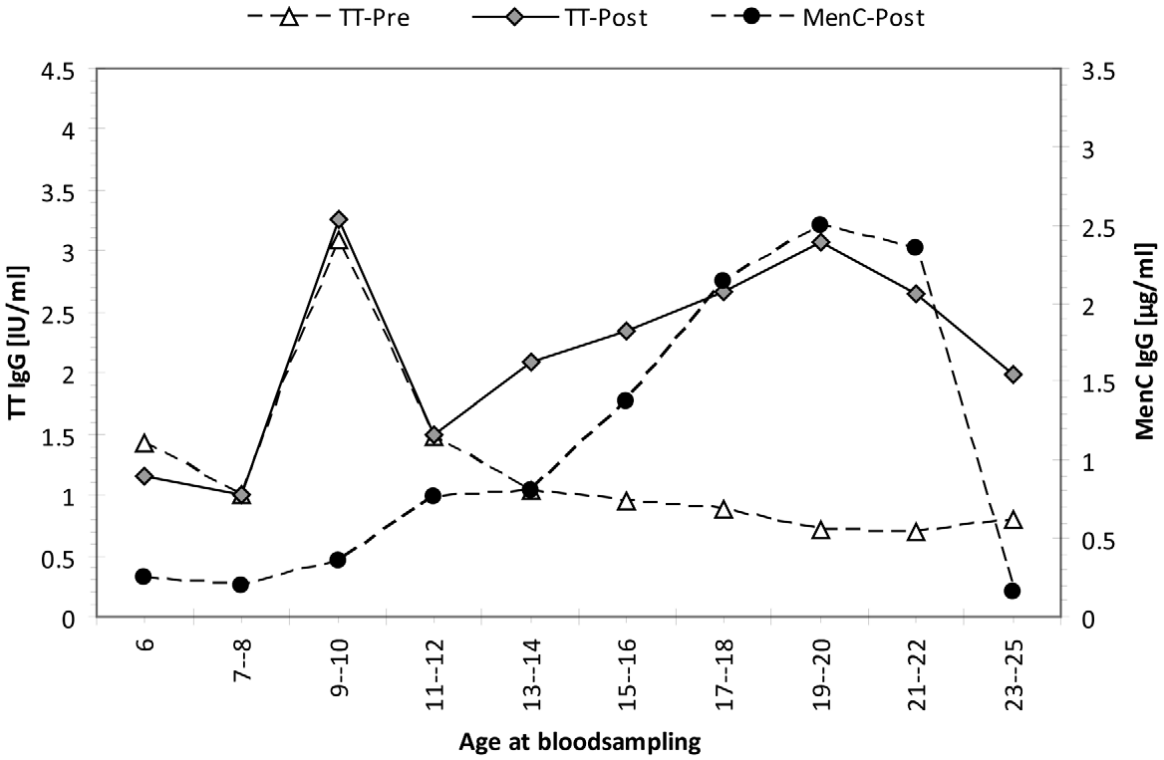
Tetanus-specific IgG antibody levels. Tetanus-specific IgG levels in the pre- and post-MenC introduction eras and MenC PS-specific IgG in the post-MenC introduction era within age-cohorts immunized during the catch-up campaign. All age cohorts were immunized in the catch-up campaign of 2002.

In children below 9 years (who did not yet receive DT-IPV booster at 9 years of age) tetanus-specific antibodies were equally high before and after introduction of MenCC vaccine. However, in the cohorts that received their MenCC immunization after the tetanus booster at 9 years of age, a clear age-related increase and persistence of tetanus-specific antibodies was observed, similar to the MenC-specific antibody levels **(** **Fig. 3** **)**.

## Discussion

The immunization strategy in the Netherlands to prevent meningococcal serogroup C disease with a single conjugate vaccination at 14 months and a large catch-up campaign for all children between 14 months and 18 years of age, has thus far been highly successful in eliminating MenC disease in all age-categories [1]. Data from the current study show that meningococcal immunization leads to improved protection with age compared to natural elicited immunity. However, serological protection rapidly wanes within a few years after a single routine immunization at 14 months of age. Immunization at an age above 5 years of age seems to result in a prolonged persistence of antibodies which gradually increases with age of immunization.

After a single vaccination at 14 months, a clear rise in IgG and bactericidal antibodies is followed by a rapid decline in antibody levels within a few years, which are comparable to levels before introduction of the vaccine. In line with this, Snape et al showed that after two years, residual bactericidal titers ≥8 remained present in only 37% of children which were immunized around 2 years of age [8]. Nonetheless, we found a higher prevalence of protective SBA titers, compared to the pre-MenC introduction era, in these age-cohorts. This relatively high bactericidal activity could furthermore almost completely be inhibited by pre-absorbing the MenC PS-specific antibodies (data not shown). This may be explained by the presence of antibodies with high avidity or it points to a shift in the induction of highly functional IgG1 antibodies compared to naturally elicited antibodies, that persist after MenCC immunization despite low titers [20], [21]. Thus, even without overall high serum IgG MenC levels, conjugate vaccination may induce ongoing protection against MenC disease.

Interestingly, we found a response that was related to the age of MenCC immunization in children who were immunized between 5 and 18 years of age in the catch-up-campaign with a gradual increase of antibody levels with age. This age-related persistence was also found in the UK, in which Snape et al [9] and Trotter and collegues [10] respectively, described a SBA prevalence of 84% and 67% in children vaccinated between 11–20 years, similar to the 77% proportion of our study population. In Greece, Sakou et al [22]. showed within the same age-group a slightly lower prevalence of 62%. Differences in the seroprevalences between studies are probably due to composition of the studies, percentage of individuals immunized in each study and the use of different meningococcal vaccines. The UK employed three different MenCC vaccines, two of which are CRM_197_ (diphtheria toxin mutant) conjugated vaccines, while only the tetanus conjugated MenC vaccine is used in the Netherlands, which is more immunogenic than the CRM_197_ conjugate vaccines [23]–[25].

The increase and persistence of circulating antibodies in the age-cohort between 5 and 18 years might be explained by nasopharyngeal carriage of, or exposure to, the meningococcal species throughout childhood and adolescence, which leads to natural priming of the maturing immune system [26]. Maturation of the immune system throughout childhood has been extensively described [27]–[29], and is suggested to be, together with ongoing exposure of the immune-system, the most likely explanation of enhanced antibody sustainment [9]. The small rise in MenC PS-specific IgG from age 5 years onwards, as also observed in the pre-MenC introduction era, underlines this maturation. However, more than 4 years after MenCC immunization the levels of circulating antibodies in this cohort are still remarkably high, while for most other vaccines multiple dosages are required to reach sustained protective levels for such a long period [30]. It may be expected that antibody titers after infant immunization are not sufficient enough to maintain seroprotection or herd-effects until adolescence. Based on the results presented here, a booster vaccination at an age just before adolescence - the age at which carriage and disease incidence were historically highest -, may be necessary to maintain protection on the long-term. Modelling studies like that of Campbell et al [31]. are very useful for the determination of the proper age at which a booster vaccination can be administered. Although due to a single adolescent immunization the transmission route for meningococci may continue to be interrupted, it is probably not preferable to move the immunization from the age of 14 months to an older age, or completely remove this immunization from the NIP, since this could potentially lead to an increase of meningococcal disease in very young children.

We observed in cohorts immunized with the MenCC vaccine between ages 10 to 18, who received a tetanus booster vaccination at 9 years of age according to the Dutch NIP, also persistent high levels of tetanus-specific antibodies compared to the levels observed in the pre-MenC introduction era. Therefore, besides the induction of immune memory by conjugate vaccination for the polysaccharide antigen [32], conjugate vaccination at later ages also induces long-lasting circulating antibodies towards the carrier protein.

We observed a complete absence of MenC PS antibodies in the first year of life before vaccination at 14 months of age, which was even lower than the levels in the pre-MenC introduction era. This is probably due to the decline in MenC PS-specific antibodies observed in the population above 25 years of age, as a result of the very low circulation of MenC after the mass immunization campaign of 2002 among the Dutch population. The lack of antibody levels in the first year of life may be compensated by maternal antibodies, but women of childbearing age also currently have low levels of MenC-specific IgG and SBA, leaving the newborns vulnerable until the first vaccination at 14 months. In the near future, when the MenCC immunized women are in the childbearing age, the protection by maternal antibodies may improve as high levels of maternal antibodies are observed in the oldest age-cohort in the catch-up campaign [33]. Nevertheless, with the estimated half-life of maternal IgG of 5 to 6 weeks, antibody levels may still not be sufficient to provide protection until the MenCC immunization at 14 months of age leaving newborns at high risk for infection if the circulation of meningococci of serogroup C increases again [34].

Non-immunized adult age-cohorts revealed distinctly lower MenC-specific IgG than before introduction of MenC vaccination. However, approximately 23% were still protected against disease based on SBA prevalence. Inhibition of MenC PS-specific antibodies revealed that this SBA result was not based on MenC PS-specific antibodies alone as was seen in vaccinated infants (data not shown). Therefore other naturally acquired antibodies are assumed to play a prominent role in the immunity of adults [35]. Due to the current low circulation of MenC, specific antibody levels may further decrease, putting these non-immunized adults at increased risk in the (near) future if MenC returns in the community.

Levels of MenY and Men W-135 PS-specific IgG were low during life, which is expected based on the low carriage and disease rates caused by these serogroups [36]. Still, with modern travelling behaviour, a large migrant population and the rise of serogroup Y in Northern America, awareness to the rise of other serogroups should be maintained. As previously also observed by Trotter et al [10], we saw increasing levels of MenA PS-specific IgG during life, despite the fact that currently only a few cases of MenA disease occur in the Netherlands. Several studies have described that polysaccharides from other bacteria may elicit cross-reactive antibodies towards the MenA PS [37]–[39], but to what extend these antibodies are functional in preventing MenA disease remains unclear.

The design of the studies ensured random selection of the subjects. The MenCC vaccination coverage in the 40 municipalities included in the serosurveillance study of 2006–2007 amounted up to 94% [3], which is similar for the overall coverage achieved in 2002. Serum samples from those who participated in the surveys are well characterized [15], [16]. From all subjects (both participants and non-participants) age and gender was known. Although men in certain age groups participated less in both surveys, we corrected for this by weighting the seroprevalence and GMT estimates according to age and gender with data from Statistics Netherlands. Furthermore persons who do not take part in the NIP, participated somewhat less in both surveys (based on the non-response questionnaire), thereby slightly overrepresenting the subgroups who have a positive attitude towards vaccination. Therefore, the seroprevalences may slightly be overestimated in the cohorts that have been eligible for MenC vaccination. However, as mentioned earlier 94% the general population was immunized in 2002 against MenC and the current coverage in the NIP is approximately 95% at 14 months, therefore little differences may be expected. In addition, we found no significant differences within the MenC eligible age-cohorts between the total sampled population and the persons for whom a MenCC immunization record was available (data not shown).

### Conclusion

A single MenCC immunization leads to improved protection compared to naturally elicited immunity. A single MenCC immunization above 5 years of age seems to induce persistent protective antibodies, whereas a single injection at 14 months leads to a rapid waning within a few years of serological protective levels. Since memory responses may not be fast enough to prevent disease, antibody persistence by vaccination seems to be the major way to prevent MenC disease [40]–[41]. If the incidence of MenC disease increases due to renewed circulation, a MenCC booster immunization just before entering adolescence may be appropriate. A booster will most likely result in a high level of protective bactericidal antibodies as previously shown in infants and adolescents [40]–[42], and will probably preserve herd-effects, which is requested for those who may be most susceptible to disease, due to a lack of antibodies (cohorts under 14 months of age). But most importantly, a second MenCC immunization will also improve protection in the vaccinated cohorts, as in the years to come a increasing part of children will be less well protected since they received only a primary immunization at 14 months.

## References

[1] de Greeff SC, de Melker HE, Spanjaard L, Schouls LM, van Derende A (2006). Protection from routine vaccination at the age of 14 months with meningococcal serogroup C conjugate vaccine in the Netherlands.. Pediatr Infect Dis J.

[2] Gezondheidsraad/Health Council of the Netherlands. (2002). Universal vaccination against meningococcal serogroup C and pneumococcal disease..

[3] Neppelenbroek SE, de Vries M, de Greeff SC, Timen A (2002). ‘da's goed gedaan? Woordverslag van de landelijke vaccinatiecampagne meningokokken C..

[4] Trotter CL, Andrews NJ, Kaczmarski EB, Miller E, Ramsay ME (2004). Effectiveness of meningococcal serogroup C conjugate vaccine 4 years after introduction.. Lancet.

[5] Larrauri A, Cano R, Garcia M, Mateo S (2005). Impact and effectiveness of meningococcal C conjugate vaccine following its introduction in Spain.. Vaccine.

[6] Cameron C, Pebody R (2006). Introduction of pneumococcal conjugate vaccine to the UK childhood immunisation programme, and changes to the meningitis C and Hib schedules.. Euro.Surveill.

[7] Richmond P, Borrow R, Goldblatt D, Findlow J, Martin S (2001). Ability of 3 different meningococcal C conjugate vaccines to induce immunologic memory after a single dose in UK toddlers.. J Infect. Dis.

[8] Snape MD, Kelly DF, Green B, Moxon ER, Borrow R (2005). Lack of serum bactericidal activity in preschool children two years after a single dose of serogroup C meningococcal polysaccharide-protein conjugate vaccine.. Pediatr.Infect.Dis J.

[9] Snape MD, Kelly DF, Lewis S, Banner C, Kibwana L (2008). Seroprotection against serogroup C meningococcal disease in adolescents in the United Kingdom: observational study.. BMJ.

[10] Trotter CL, Borrow R, Findlow J, Holland A, Frankland S (2008). Seroprevalence of antibodies against serogroup C meningococci in England in the postvaccination era. Clin. Vaccine Immunol.

[11] Borrow R, Andrews N, Goldblatt D, Miller E (2001). Serological basis for use of meningococcal serogroup C conjugate vaccines in the United Kingdom: reevaluation of correlates of protection.. Infect Immun.

[12] Borrow R, Balmer P, Miller E (2005). Meningococcal surrogates of protection--serum bactericidal antibody activity.. Vaccine.

[13] Backhouse JL, Gidding HF, MacIntyre CR, McIntyre PB, Gilbert GL (2007). Population-based seroprevalence of Neisseria meningitidis serogroup C capsular antibody before the introduction of conjugate vaccine, in Australia.. Vaccine.

[14] Ceyhan M, Yildirim I, Balmer P, Riley C, Laher G (2007). Age-specific seroprevalence of serogroup C meningococcal serum bactericidal antibody activity and serogroup A, C, W135 and Y-specific IgG concentrations in the Turkish population during 2005.. Vaccine.

[15] de Melker HE, Conyn-van Spaendonck MA (1998). Immunosurveillance and the evaluation of national immunization programmes: a population-based approach.. Epidemiol.Infect.

[16] van der Klis FR, Mollema L, Berbers GA, de Melker HE, Coutinho RA (2009). Second national serum bank for population-based seroprevalence studies in the Netherlands.. Neth J Med.

[17] de Voer RM, Schepp RM, Versteegh FG, van der Klis FR, Berbers GA (2009). Simultaneous detection of Haemophilus influenzae type b polysaccharide-specific antibodies and Neisseria meningitidis serogroup A, C, Y, and W-135 polysaccharide-specific antibodies in a fluorescent-bead-based multiplex immunoassay.. Clin.Vaccine Immunol.

[18] van Gageldonk PG, van Schaijk FG, van der Klis FR, Berbers GA (2008). Development and validation of a multiplex immunoassay for the simultaneous determination of serum antibodies to Bordetella pertussis, diphtheria and tetanus.. J Immunol Methods.

[19] Maslanka SE, Gheesling LL, Libutti DE, Donaldson KB, Harakeh HS (1997). Standardization and a multilaboratory comparison of Neisseria meningitidis serogroup A and C serum bactericidal assays. The Multilaboratory Study Group.. Clin Diagn Lab Immunol.

[20] Goldblatt D, Vaz AR, Miller E (1998). Antibody avidity as a surrogate marker of successful priming by Haemophilus influenzae type b conjugate vaccines following infant immunization.. J Infect Dis.

[21] Bredius RG, Driedijk PC, Schouten MF, Weening RS, Out TA (1992). Complement activation by polyclonal immunoglobulin G1 and G2 antibodies against Staphylococcus aureus, Haemophilus influenzae type b, and tetanus toxoid.. Infect Immun.

[22] Sakou II, Tzanakaki G, Tsolia MN, Sioumala M, Barbouni A (2009). Investigation of serum bactericidal activity in childhood and adolescence 3-6 years after vaccination with a single dose of serogroup C meningococcal conjugate vaccine.. Vaccine.

[23] Southern J, Borrow R, Andrews N, Morris R, Waight P (2009). Immunogenicity of a reduced schedule of meningococcal group C conjugate vaccine given concomitantly with the Prevenar and Pediacel vaccines in healthy infants in the United Kingdom.. Clin Vaccine Immunol.

[24] Díez-Domingo J, Planelles Cantarino MV, Baldó Torrentí JM, Sansano MI, Rosich AJ (2010). A Randomized, Multicenter, Open-Label Clinical Trial to Assess the Immunogenicity of a Meningococcal C Vaccine Booster Dose Administered to Children Aged 14 to 18 Months.. Pediatr Infect Dis J.

[25] Borrow R, Andrews N, Findlow H, Waight P, Southern J (2010). Kinetics of antibody persistence following administration of a combination meningococcal serogroup C and haemophilus influenzae type b conjugate vaccine in healthy infants in the United Kingdom primed with a monovalent meningococcal serogroup C vaccine.. Clin Vaccine Immunol.

[26] Goldschneider I, Gotschlich EC, Artenstein MS (1969). Human immunity to the meningococcus. I. The role of humoral antibodies.. J Exp.Med.

[27] Weill JC, Weller S, Reynaud CA (2009). Human marginal zone B cells.. Annu Rev Immunol.

[28] Upham JW, Lee PT, Holt BJ, Heaton T, Prescott SL (2002). Development of interleukin-12-producing capacity throughout childhood.. Infect Immun.

[29] Pihlgren M, Friedli M, Tougne C, Rochat AF, Lambert PH (2006). Reduced ability of neonatal and early-life bone marrow stromal cells to support plasmablast survival.. J Immunol.

[30] Feiring B, Fuglesang J, Oster P, Naess LM, Helland OS (2006). Persisting immune responses indicating long-term protection after booster dose with meningococcal group B outer membrane vesicle vaccine.. Clin Vaccine Immunol.

[31] Campbell H, Andrews N, Borrow R, Trotter C, Miller E (2010). Updated postlicensure surveillance of the meningococcal C conjugate vaccine in England and Wales: effectiveness, validation of serological correlates of protection, and modeling predictions of the duration of herd immunity.. Clin Vaccine Immunol.

[32] Blanchard RG, Snape MD, Kelly DF, John T, Morant A (2008). The magnitude of the antibody and memory B cell responses during priming with a protein-polysaccharide conjugate vaccine in human infants is associated with the persistence of antibody and the intensity of booster response.. J Immunol.

[33] de Voer RM, van der Klis FR, Nooitgedagt JE, Versteegh FG, van Huisseling JC (2009). Seroprevalence and placental transportation of maternal antibodies specific for Neisseria meningitidis serogroup C, Haemophilus influenzae type B, diphtheria, tetanus, and pertussis.. Clin Infect Dis.

[34] O'Dempsey TJ, McArdle T, Ceesay SJ, Secka O, Demba E (1996). Meningococcal antibody titres in infants of women immunised with meningococcal polysaccharide vaccine during pregnancy.. Arch Dis Child Fetal Neonatal Ed.

[35] Goldschneider I, Gotschlich EC, Artenstein MS (1969). Human immunity to the meningococcus. II. Development of natural immunity.. J Exp Med.

[36] Maiden MC, Stuart JM (2002). Carriage of serogroup C meningococci 1 year after meningococcal C conjugate polysaccharide vaccination.. Lancet.

[37] Robbins JB, Myerowitz L, Whisnant JK, Argaman M, Schneerson R (1972). Enteric bacteria cross-reactive with Neisseria meningitidis groups A and C and Diplococcus pneumoniae types I and 3.. Infect Immun.

[38] Myerowitz RL, Gordon RE, Robbins JB (1973). Polysaccharides of the genus Bacillus cross-reactive with the capsular polysaccharides of Diplococcus pneumoniae type 3, Haemophilus influenzae type b, and Neisseria meningitidis group A.. Infect Immun.

[39] Vann WF, Liu TY, Robbins JB (1976). Bacillus pumilus polysaccharide cross-reactive with meningococcal group A polysaccharide.. Infect Immun.

[40] Snape MD, Kelly DF, Salt P, Green S, Snowden C (2006). Serogroup C meningococcal glycoconjugate vaccine in adolescents: persistence of bactericidal antibodies and kinetics of the immune response to a booster vaccine more than 3 years after immunization.. Clin Infect Dis.

[41] de Voer RM, van der Klis FR, Engels CW, Schepp RM, van de Kassteele J (2009). Kinetics of antibody responses after primary immunization with meningococcal serogroup C conjugate vaccine or secondary immunization with either conjugate or polysaccharide vaccine in adults.. Vaccine.

[42] Richmond P, Borrow R, Miller E, Clark S, Sadler F (1999). Meningococcal serogroup C conjugate vaccine is immunogenic in infancy and primes for memory.. J Infect Dis.

